# The Effects of Probiotic–Prebiotic Use on Acute Inflammatory Parameters in Patients Receiving Pelvic Radiotherapy: A Prospective Multicenter Study

**DOI:** 10.1155/mi/1441225

**Published:** 2025-07-13

**Authors:** Muge Akmansu, Sefika Dincer, Esra Kaytan Saglam, Zuleyha Akgun, Hatice Pasaoglu

**Affiliations:** ^1^Department of Radiation Oncology, Gazi University Hospital, Ankara, Türkiye; ^2^Department of Radiation Oncology, Institute of Oncology: Istanbul, Istanbul, Türkiye; ^3^Department of Radiation Oncology, Uskudar University Hospital, Istanbul, Türkiye; ^4^Department of Clinical Biochemistry, Gazi University Hospital, Ankara, Türkiye

## Abstract

**Background:** This is the first prospective clinical study to evaluate the impact of prebiotic and probiotic use on acute radiation-induced pelvic side effects and associated changes in serum cytokine levels in patients with pelvic malignancies.

**Methods:** This multicenter prospective study included 80 patients with pelvic cancers treated at two centers between 2021 and 2023. Patients were divided into three groups: probiotic + prebiotic, prebiotic only, and nonsupplemented. Serum cytokine levels (tumor necrosis factor-alpha (TNF-α) and interleukin (IL)-6) and radiation-related side effect scores were assessed at the beginning of pelvic radiotherapy (RT) and again at the fifth week. Based on data normality, the Wilcoxon signed-rank test was used for statistical analysis.

**Results:** According to side effect scores, in the prebiotic-only group, a modest yet statistically significant increase in fecal incontinence (FI; *p*=0.040) and blood and mucus in the stool (BMS; *p*=0.049) was observed. Conversely, the probiotic + prebiotic group showed a significant increase in dysuria (*p*=0.001) and impotence (*p*=0.016). IL-6 levels significantly increased in the prebiotic-only group (*p*=0.035), while TNF-α levels rose significantly in the group without any nutritional support (*p*=0.025). No significant increase in cytokine levels or intestinal side effects was observed in the combined probiotic + prebiotic group (*p*  > 0.05 for all comparisons).

**Conclusions:** The absence of a rise in cytokine levels and intestinal toxicity in the probiotic + prebiotic group suggests that probiotics may play a role in mitigating radiation-induced intestinal side effects and inflammatory responses.

## 1. Introduction

Pelvic malignancies significantly affect the elderly population. Prostate cancer is the most frequently diagnosed cancer among men, while rectal cancer remains a leading cause of cancer-related morbidity and mortality worldwide [1]. Both require complex and multidisciplinary treatment strategies to achieve optimal outcomes. Among these, radiation therapy has become a cornerstone for local tumor in the nonsupplemented group and improved survival.

However, the benefits of pelvic radiotherapy (RT) are often accompanied by acute treatment-related side effects, including diarrhea, rectal bleeding, abdominal discomfort, and urinary complications [2, 3]. These adverse effects, particularly prevalent in patients with prostate, rectal, and other pelvic cancers, can impair quality of life. Symptom severity is typically assessed using tools such as the Bristol Stool Scale (BSS) [4] and the European Organization for Research and Treatment of Cancer Quality of Life Questionnaire-Colorectal Cancer 29 (EORTC QLQ-CR29) [5, 6].

The interaction between gut microbiota and radiation therapy has gained increasing attention. Radiation can disrupt the intestinal microbiome, potentially worsening treatment-related toxicities [7]. In contrast, dietary fibers, particularly prebiotics, can positively modulate the gut microbiota, thereby increasing the production of short-chain fatty acids (SCFAs) [8], which play a crucial role in maintaining mucosal health. This suggests that prebiotic and probiotic supplementation may help mitigate radiation-induced intestinal toxicity.

Radiation-related mucosal inflammation is driven in part by the activation of nuclear factor kappa B (NF-*κ*B), a transcription factor that regulates proinflammatory cytokines and chemokines such as tumor necrosis factor-alpha (TNF-α), interleukin (IL)-1, IL-2, and IL-6 [9–11]. Prior clinical studies have associated radiation-induced tissue damage with elevated serum levels of IL-1β, TNF-α, and IL-6 in patients with pelvic cancers [12–14]. Accordingly, changes in these cytokines may serve as biomarkers of acute radiation toxicity.

To our knowledge, this is the first prospective study to evaluate the relationship between probiotic–prebiotic supplementation, radiation-induced pelvic side effects, and changes in serum cytokine levels in patients with pelvic malignancies.

## 2. Methods

### 2.1. Patient Accrual

This prospective study enrolled 80 patients with primary pelvic malignancies treated with pelvic RT at two centers between December 2021 and July 2023. Twenty-four patients were treated at Center A and 56 at Center B. All patients at Center A received combined probiotic and prebiotic supplementation. Patients at Center B were assigned to one of three groups: probiotic + prebiotic, prebiotic only, or nonsupplemented group.

Inclusion criteria were: Diagnosis of primary pelvic cancer (prostate, rectal, endometrial, cervical, or bladder), indication for pelvic RT, Eastern Cooperative Oncology Group (ECOG) performance status 0–2, and ability to take oral supplements. Exclusion criteria included history of malignancies other than breast or thyroid cancer, ECOG score of 3–4, or presence of a colostomy.

All participants provided written informed consent. The study was approved by the Ethics Committee of Center A.

### 2.2. Pelvic RT

All patients underwent computed tomography (CT)–based simulation in the supine position with a slice thickness of 3–4 mm. RT was delivered using linear accelerators with a 6 MV photon energy, employing intensity-modulated radiotherapy (IMRT). Treatment was administered 5 days a week with total doses ranging from 45 to 76 Gy, using standard fractionation (1.8–2 Gy per fraction) to the planning target volume (PTV). Target delineation followed the Radiation Therapy Oncology Group (RTOG) contouring guidelines [15]. Cone-beam CT was used for daily image guidance to ensure accurate setup.

### 2.3. Side Effect Evaluation

Radiation-related side effects were assessed using two validated tools: the EORTC QLQ-CR29 and the BSS. The EORTC QLQ-CR29 includes five functional and 18 symptom scales, evaluating various domains such as urinary frequency (UF), blood and mucus in stool (BMS), stool frequency (SF), body image (BI), and other symptoms including dysuria, abdominal pain, flatulence, fecal incontinence (FI), and sexual functioning. Scores range from 0 to 100, with higher scores indicating either more severe symptoms or better functioning, depending on the specific scale. The BSS is a seven-point ordinal scale used to classify stool consistency. Types 1 and 2 indicate constipation, while Types 6 and 7 suggest diarrhea. Types 3–5 are considered within the normal range. Patients were asked to complete both assessment tools at baseline (Week 1) and again during the fifth week of pelvic RT.

### 2.4. Proinflammatory Cytokine Assessment

Venous blood samples were collected during the first and fifth weeks of RT. Following collection, blood was allowed to clot at room temperature for 30 min, then, centrifuged at 3000 rpm for 10 min. The serum was aliquoted into transfer tubes and stored at −70°C until analysis.

Serum levels of IL-6 and TNF-α were measured using a standardized human immunoassay kit. All samples were processed using the same protocol. Due to possible intercenter variability, cytokine data from Center A were analyzed separately.

### 2.5. Statistical Analysis

Cytokine levels and symptom scores from Weeks 1 to 5 were analyzed within each group. Because baseline cytokine reference ranges differed between centers, IL-6 and TNF-α levels were evaluated separately per center.

The Wilcoxon signed-rank test was used for within-group comparisons. Subgroup analyses were also conducted based on cancer type and supplementation status. All analyses were performed using IBM SPSS Statistics (version 29.0.1). A *p*-value of <0.05 was considered statistically significant.

Detailed statistical outputs and extended results can be found in the Supporting Information.

## 3. Results

### 3.1. Patient Characteristics

The mean age of the study population was 65.0 years (range: 40–82 years). Body mass index (BMI) ranged from 17 to 35 kg/m^2^. The cohort included five types of pelvic malignancies: prostate, rectal, endometrial, cervical, and bladder cancers. A detailed breakdown is provided in Table 1.

### 3.2. Serum Cytokine Levels

In the group without nutritional support, serum TNF-α levels increased significantly from baseline to Week 5 (*p*=0.025). Among patients receiving only prebiotics, TNF-α levels did not change significantly; however, IL-6 levels increased significantly (*p*=0.035). In contrast, no significant changes were observed in either TNF-α or IL-6 levels in the probiotic + prebiotic group across both centers (*p*  > 0.05 for all comparisons; Table 2).

Additionally, in prostate cancer patients, the probiotic group demonstrated a significant decrease in prostate-specific antigen (PSA) levels between Weeks 1 and 5 (*p*=0.008). A similar reduction was observed in the prebiotic group (*p*=0.021).

### 3.3. Clinical Symptom Scaling

Side effect scores obtained using the EORTC QLQ-CR29 and BSS were compared between Weeks 1 and 5. In the probiotic + prebiotic group, significant increases were observed in dysuria scores (*p*=0.001) and impotence scores (*p*=0.016). In the prebiotic-only group, small but statistically significant increases were noted in FI scores (*p*=0.040) and BMS scores (*p*=0.049). Detailed results are presented in Tables 3 and 4.

### 3.4. Subgroup Analysis

Subgroup analyses were conducted based on cancer type, specifically comparing prostate and rectal cancers, as well as supplementation status, including nonsupplemented group, prebiotic-only, and probiotic + prebiotic groups. Among prostate cancer patients receiving combined probiotic and prebiotic support, dysuria scores increased from 7.88 ± 18.29 to 18.28 ± 18.29 (*p*=0.037), while impotence scores rose from 62.66 ± 31.21 to 75.99 ± 18.87 (*p*=0.014). In contrast, no statistically significant changes in symptom scores were observed in the probiotic + prebiotic and prebiotic-only groups with rectal cancer, although there were numerical increases in bowel symptom scores. Regarding cytokine levels, the prostate cancer nonsupplemented group exhibited a significant posttreatment increase in TNF-α levels (*p*=0.036), while IL-6 levels remained unchanged. No statistically significant cytokine changes were observed in other subgroups, including all rectal cancer groups.

## 4. Discussion

This study investigated the effects of probiotic and prebiotic supplementation on inflammatory cytokine changes and radiation-induced intestinal side effects in patients undergoing pelvic RT. The findings suggest that adding probiotics to prebiotic supplementation may help mitigate radiation-related intestinal toxicity and modulate immune responses by preventing significant increases in proinflammatory cytokines, such as IL-6 and TNF-α [16, 17]. This suggests a potential protective role of combined probiotic and prebiotic supplementation in reducing radiation-induced inflammation and gastrointestinal side effects, contributing to the growing body of evidence supporting microbiota-targeted interventions for improving radiation therapy outcomes.

Disruptions in gut microbiota due to radiation have been linked to increased expression of TNF-α and IL-1β, reinforcing the role of the gut–immune axis in radiation toxicity. Clinical studies also report elevated IL-6 levels in patients with Grade 2 diarrhea during pelvic radiation therapy, whereas levels of IL-1β and TNF-α remain unchanged. Similarly, the elevated IL-6 levels in the prebiotic-only group and increased TNF-α in the nonsupplemented group in our study may support this immune axis and proinflammatory response.

In contrast, the probiotic + prebiotic group in our study did not exhibit significant increases in serum IL-6 or TNF-α levels, nor did it show an increase in radiation-related gastrointestinal symptoms. These results indicate a potential synergistic effect of probiotics in modulating immune responses and protecting against radiation-induced mucosal damage. Probiotics have been shown to attenuate radiation-induced gut toxicity by enhancing mucosal barrier integrity, reducing the production of inflammatory cytokines, and promoting the colonization of beneficial microorganisms. These findings suggest that probiotic supplementation may counteract the proinflammatory effects of radiation by modulating gut microbiota composition and function [12–14].

Although the changes in inflammatory cytokines may initially appear to reflect the immune axis, they could be attributed to different underlying mechanisms. Another notable observation was the increased incidence of urological symptoms in patients consuming probiotics and prebiotics despite the absence of significant cytokine alterations in this group. This finding may be explained by gut–urinary axis interactions, where microbial changes in the gut influence local immune responses in the urinary tract [18, 19]. Gut-derived metabolites, including SCFAs, influence bladder epithelial integrity, and immune responses. Previous research has demonstrated that SCFAs can regulate epithelial permeability and immune activation, potentially contributing to dysuria or urinary discomfort even in the absence of systemic cytokine elevation [20–22]. This highlights the need for further investigation into the systemic effects of gut microbiota modulation, extending beyond its impact on the intestinal environment alone. Future studies should focus on identifying the specific microbial and metabolic pathways influencing urinary symptoms, incorporating urinary biomarkers and microbiome profiling to better characterize the systemic effects of gut microbiota modulation in the context of radiation therapy.

The observed increase in IL-6 levels without a corresponding rise in TNF-α among patients receiving only prebiotics may be attributed to the selective immunomodulatory effects of prebiotics. Prebiotics enhance the production of SCFAs, which modulate regulatory T-cell activity and favor IL-6 secretion while exerting minimal effects on TNF-α [23, 24]. Furthermore, IL-6 plays dual roles in both proinflammatory and anti-inflammatory pathways; the increase in IL-6 observed in this study may reflect an adaptive and anti-inflammatory response rather than overt inflammation.

A key concern raised in the review process was the relevance of PSA measurements in this study. While PSA was initially considered a secondary endpoint, its fluctuations are heavily influenced by androgen deprivation therapy (ADT), making it difficult to distinguish whether changes are due to probiotic–prebiotic supplementation, tumor regression, or prostatitis. ADT significantly reduces PSA levels, independent of inflammation, which poses a confounding factor. Additionally, radiation-induced prostatitis can lead to transient PSA increases, further complicating its interpretation. Future studies should investigate alternative inflammatory markers for prostatitis that are less susceptible to hormonal therapy [25, 26].

The study cohort included patients with prostate, rectal, and other pelvic malignancies, introducing variability in treatment responses. While this heterogeneity enhances the generalizability of findings, it also presents challenges in identifying subgroup-specific effects. To address this, subgroup analyses were performed, stratifying patients based on cancer type. In the prostate cancer cohort, the probiotic + prebiotic group exhibited a significant increase in dysuria scores (*p*=0.037) and impotence scores (*p*=0.014). These findings suggest a potential interaction between microbiota modulation and urinary tract responses, particularly in patients with prostate cancer. Notably, the prostate cancer nonsupplemented subgroup showed a significant posttreatment increase in TNF-α levels (*p*=0.036). In contrast, the other subgroups did not exhibit statistically significant changes in IL-6 or TNF-α levels, suggesting a possible protective effect of probiotics and prebiotics against radiation-induced inflammation. The absence of significant differences in the remaining groups may be attributed to the relatively small sample size, which could have limited the statistical power to detect subtle changes. These findings underscore the need for more comprehensive, well-powered studies to confirm these associations and to investigate further the mechanisms underlying the differential responses observed among subgroups.

This study has several strengths, including its prospective design, well-defined patient groups, and integration of clinical and cytokine–based assessments. It provides valuable insights into the effects of probiotic and prebiotic supplementation on inflammatory responses and radiation-induced side effects. However, certain limitations should be acknowledged.

The analysis did not account for other potential factors that may influence the proinflammatory process, such as chemotherapy, bacterial infections, and rheumatological diseases. Additionally, to better evaluate the effects of probiotic–prebiotic supplementation on intestinal side effects, future studies should include a side effect scoring system for patients without nutritional support, enabling more evident comparisons. Moreover, the small sample size and lack of long-term follow-up may limit the generalizability of the findings.

Another limitation is the reliance on systemic cytokine measurements, which may have overlooked localized inflammatory responses within the intestinal and urinary tracts. Future research should incorporate broader biomarker panels, such as gut microbiota profiling, fecal calprotectin, and urinary metabolomic analyses, to provide a more comprehensive understanding of microbiota–host interactions in the context of radiation therapy. Expanding the study design to include larger cohorts and extended follow-up periods will be essential to validate these findings and optimize microbiota-targeted interventions for managing radiation-induced toxicities.

Future perspectives include expanding study populations to better evaluate the long-term impact of probiotic and prebiotic supplementation on inflammation and clinical outcomes in pelvic radiation therapy patients. Advanced metabolomic and microbiome analyses should be incorporated to elucidate underlying mechanisms and identify microbial signatures predictive of treatment response. Additionally, exploring alternative inflammatory biomarkers beyond cytokine levels, such as fecal calprotectin or urinary metabolite profiles, may provide a more comprehensive assessment of inflammation [27]. Identifying specific microbial strains that optimize therapeutic outcomes could pave the way for personalized microbiota-targeted interventions in oncology care. Further investigation into the role of probiotics and prebiotics in mitigating radiation-induced toxicity and preserving gut barrier function through clinical trials with nonsupplemented groups will be essential for translating these findings into clinical practice.

## 5. Conclusion

In conclusion, this study provides evidence that probiotic supplementation may reduce radiation-induced intestinal toxicity and modulate inflammatory responses in patients undergoing pelvic radiation therapy. The differential cytokine and clinical responses observed between the prebiotic-only and probiotic + prebiotic groups underscore the complexity of gut–microbiota interactions in radiation therapy. However, cytokine changes may be influenced by different underlying mechanisms, highlighting the need for further investigations. Additionally, an increased incidence of dysuria was observed in the probiotic + prebiotic group, suggesting a possible interaction between supplementation and urinary tract responses. The underlying mechanism of this association remains unclear and warrants further investigation in larger-scale studies that incorporate comprehensive side effect assessments. Nonetheless, probiotic supplementation appears to be a well-tolerated option for patients undergoing pelvic RT and may offer a promising supportive strategy in managing treatment-related toxicities.

## Figures and Tables

**Table 1:** Primary cancer sites.

Primary cancer site	Prebiotic + probiotic (*N* (%))	Only-prebiotic (*N* (%))	No dietary supplement (*N* (%))
Prostate	16 (42)	9 (41)	8 (40)
Rectal	14 (37)	9 (41)	6 (30)
Uterine	3	2	3
Cervix	3	1	2
Anorectal	1	—	—
Bladder	1	1	1

**Table 2:** Serum cytokine and PSA levels (mean value ± SD).

	Probiotic + prebiotic (*N* = 14 (24)^a^)	*p* value	Only-prebiotic (*N* = 22)	*p* value	Nonsupplemented (*N* = 20)	*p* value
TNF-α (pg/mg) 1 w 5 w	12.03 ± 5.61 (3.83 ± 2.37^a^) 9.03 ± 3.00 (3.77 ± 2.29^a^)	0.055 (0.906^a^)	9.75 ± 2.27 9.35 ± 2.38	0.38	7.31 ± 4.76 9.51 ± 3.75	**0.025**
IL-6 (pg/mg) 1 w 5 w	12.08 ± 14.70 (7.01 ± 6.15^a^) 9.20 ± 10.81 (7.56 ± 6.39^a^)	0.158 (0.115^a^)	7.03 ± 8.95 11.68 ± 14.82	**0.035**	10.42 ± 11.91 12.34 ± 11.20	0.42
PSA (ng/mL) 1 w 5 w	3.07 ± 5.55 0.76 ± 1.67	**0.008**	3.41 ± 4.60 1.90 ± 3.26	**0.021**	7.01 ± 12.60 2.29 ± 5.24	**0.028**

*Note:* TNF < 12.4 (standard reference value); IL-6 < 7 (standard reference value). *N*, number of patients. Bold values indicate statistically significant differences (*p* < 0.05).

Abbreviations: IL-6, interleukin-6; PSA, prostate-specific antigen; TNF-α, tumor necrosis factor-alpha; w, week.

^a^Institude-A: TNF-α and IL-6 of each center were evaluated statistically separately (TNF < 12.2 [standard reference value], IL-6 < 5.9 [standard reference value]).

**Table 3:** Clinical symptom scaling (prebiotic + probiotic).

Probiotic + prebiotic (*N* = 38)	Mean	*p*
DY-1 DY-5	%7.8840 %18.2855	**0.001**
IMP-1(*N* = 28) IMP-5	%62.6648 %75.9976	**0.016**

*Note:* “-1”, first week; ”-5”, fifth week. Bold values indicate statistically significant differences (*p* < 0.05).

Abbreviations: DY, dysuria; IMP, impotence.

**Table 4:** Clinical symptom scaling (prebiotic).

Prebiotic (*N* = 22)	Mean	*p*
BMS-1 BMS-5	7.5820 14.7029	**0.049**
FI-1 FI-5	1.7542 9.8029	**0.040**
BSS-1 BSS-5	4.2500 4.7778	0.184

*Note*: Bold values indicate statistically significant differences (*p* < 0.05).

Abbreviations: BMS, blood and mucus in stool; BSS, Bristol Stool Scale; FI, fecal incontinence.

## Data Availability

The data that support the findings of this study are included in the supporting information of this article.

## Supporting Information

Supporting Information Additional tables, extended statistical analyses, and subgroup results are provided in the supporting information.
