# Supplementary material for: The Effects of Probiotic–Prebiotic Use on Acute Inflammatory Parameters in Patients Receiving Pelvic Radiotherapy: A Prospective Multicenter Study
Source: Mediators Inflamm. 2025 Jul 13;2025:1441225. doi: 10.1155/mi/1441225 (PMC12277052; doi:10.1155/mi/1441225)
Supplement: Supporting Information — Additional tables, extended statistical analyses, and subgroup results are provided in the supporting information. [file 1441225.f1.pdf]

Clinical Symptom Scaling

(Prebiotic(N=22))	mean	p	n
**UF-1**	46,823	0,245	22
**UF-5**	53,9176		
**UI-1**	14,9985	0,081	22
**UI-5**	21,5671		
**DY-1**	11,1105	0,091	22
**DY-5**	23,5282		
**AP-1**	22,2205	0,655	22
**AP-5**	25,4882		
**BP-1**	18,3315	0,128	22
**BP-5**	27,4488		
**BF-1**	21,6650	0,949	22
**BF-5**	21,5665		
**BMS-1**	7,5820	**0,049**	22
**BMS-5**	14,7029		
**DM-1**	33,3305	0,274	22
**DM-5**	27,4488		
**HL-1**	8,3325	0,905	22
**HL-5**	5,8818		
**TA-1**	9,9990	0,381	22
**TA-5**	5,8818		
**FL-1**	24,5600	0,212	22
**FL-5**	17,6459		
**FI-1**	1,7542	**0,040**	22
**FI-5**	9,8029		
**SS-1**	6,6660	0,152	22
**SS-5**	15,6853		
**SF-1**	12,2789	0,102	22
**SF-5**	26,4682		
**EMB-1**	15,7879	0,082	22
**EMB-5**	27,4688		
**ANX-1**	81,6640	**0,002**	22
**ANX-5**	62,4944		
**WEI-1**	91,6650	0,298	22
**WEI-5**	95,8325		

**BI-1**	88,8855	0,365	22
**BI-5**	88,1913		
**IMP-1**	44,4433	0,066	6
**IMP-5**	77,7200		
**SEXM-1**	46,6640	0,257	5
**SEXM-5**	19,9980		
**DYS-1**	13,3320	1,000	5
**DYS-5**	20,0000		
**SEXW-1**	24,9975	0,180	4
**SEXW-5**	0,000		
**Bristol-1**	4,2500	0,184	22
**Bristol-5**	4,7778		

BMS: blood and mucus in stool, FI: fecal incontinence

Clinical Symptom Scaling

(Prebiotic+Probiotic(N=38))	mean	p
**UF-1**	%41,062	0,064
**UF-5**	%46,4975	
**UI-1**	%12,8459	0,777
**UI-5**	%13,5569	
**DY-1**	%7,8840	**0,001**
**DY-5**	%18,2855	
**AP-1**	%16,5256	0,694
**AP-5**	%17,1941	
**IMP-1(N=28)**	%62,6648	**0,016**
**IMP-5**	%75,9976	
**SEXM-1(N=28)**	%23,9848	0,586
**SEXM-5**	%21,3316	
**BP-1**	%16,6650	0,388
**BP-5**	%12,8192	
**BF-1**	%14,5028	0,067
**BF-5**	%10,2562	
**BMS-1**	%7,07	1,000
**BMS-5**	%7,69	
**DM-1**	%26,6	0,592
**DM-5**	%28,2031	
**HL-1**	%0	1,000
**HL-5**	%0	
**TA-1**	%4,1352	0,219
**TA-5**	%2,5638	
**FL-1**	%10,2556	0,837
**FL-5**	%15,3831	
**FI-1**	%4,7005	0,388
**FI-5**	%10,2554	
**SS-1**	%4,6315	0,733

**SS-5**	%7,6915	
**SF-1**	%16,0229	0,284
**SF-5**	%11,5354	
**EMB-1**	%18,3751	0,674
**EMB-5**	%17,1238	
**ANX-1**	%62,5738	0,448
**ANX-5**	%69,2282	
**WEI-1**	%89,9034	0,673
**WEI-5**	%85,9483	
**BI-1**	%82,7171	0,059
**BI-5**	%73,4309	
**Bristol-1**	4,0385	0,590
**Bristol-5**	3,9231	

Abbreviation: “-1”, first week;”-5”, fifth week.

Subscales: UF = urinary frequency; BMS = blood and mucus in stool; SF = stool frequency; BI = body image; DSP = defecation/stoma problemsSingle items: UI = urinary incontinence; DY = dysuria; AP = abdominal pain; BP = buttock pain; BF = bloating; DM = dry mouth; HL = hair loss; TA = taste; ANX = anxiety; WEI = weight; FL = flatulence; FI = fecal incontinence; SS = sore skin; EMB = embarrassment; STO = stoma care problems; IMP = impotence; DYS = dyspareunia; SEXM = sexual interest (men); SEMW = sexual interest (women)
